# Development and Validation of a Multiparametric Semiquantitative Scoring System for the Histopathological Assessment of Ischaemia Severity in Skeletal Muscle

**DOI:** 10.1155/2023/5592455

**Published:** 2023-03-16

**Authors:** Clara Sanz-Nogués, Michael Creane, Sean O. Hynes, Xizhe Chen, Christine Ayu Lagonda, Katarzyna Goljanek-Whysall, Timothy O'Brien

**Affiliations:** ^1^Regenerative Medicine Institute (REMEDI), Biomedical Science Building, University of Galway (Ireland), Galway, Ireland; ^2^CÚRAM SFI Research Centre for Medical Devices, University of Galway (Ireland), Galway, Ireland; ^3^Discipline of Pathology, University of Galway (Ireland), Galway, Ireland; ^4^Division of Anatomic Pathology, University Hospital Galway, Galway, Ireland; ^5^Department of Physiology, School of Medicine, University of Galway (Ireland), Galway, Ireland

## Abstract

Skeletal muscle is one of the most abundant and dynamic tissues of the body, with a strong regenerative capacity. Muscle injuries can occur as a result of a variety of events, including tissue ischaemia. Lower limb ischaemia occurs when there is an insufficient nutrient and oxygen supply, often caused by stenosis of the arteries due to atherosclerosis. The aim of this study was to develop and validate a multiparametric scoring tool for assessing ischaemia severity in skeletal muscle in a commonly used preclinical animal model. Tissue ischaemia was surgically induced in mice by ligation and excision of the femoral artery. Calf muscles were carefully dissected, prepared for histological analysis, and scored for inflammation, fibrosis, necrosis, adipocyte infiltration, and muscle fibre degeneration/regeneration. Kendall's coefficient of concordance (*W*) showed a very good agreement between the appraisers when scoring each individual histological feature: inflammation (*W* = 0.92, *p* ≤ 0.001), fibrosis (*W* = 0.94, *p* ≤ 0.001), necrosis (*W* = 0.77, *p* ≤ 0.001), adipocyte infiltration (*W* = 0.91, *p* ≤ 0.001), and fibre degeneration/regeneration (*W* = 0.86, *p* ≤ 0.001). Intrarater agreement was also excellent (*W* = 0.94 or more, *p* ≤ 0.001). There was a statistically significant negative association between the level of muscle ischaemia damage and the calf muscle weight and skeletal muscle fibre diameter. Here, we have developed and validated a new multiparametric, semiquantitative scoring system for assessing skeletal muscle damage due to ischaemia, with excellent inter- and intrarater reproducibility. This scoring system can be used for assessing treatment efficacy in preclinical models of hind limb ischaemia.

## 1. Background

**Figure 1:**
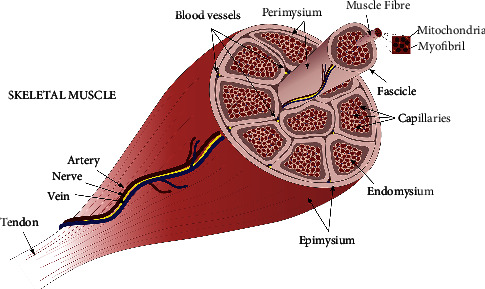
Structure of skeletal muscle. The epimysium is a tough connective tissue that surrounds bundles of long muscle fibers called muscle fascicles. These fascicles are surrounded by their own protective layer called the perimysium. The fascicles contain a bundle of muscle fibres that are wrapped a thin layer of connective tissue called endomysium. The nuclei of myofibers are oval-shaped and are located at the periphery of the cell. Each myofiber contain multiple myofibrils, which are composed of numerous sarcomeres, the smallest functional unit of a skeletal muscle fiber, and have light and dark regions that give the cell its striated appearance. Myofibers also have numerous mitochondria for energy generation. The deep neurovascular bundle is a structure of protective connective tissue that surrounds nerves, artery, and veins, so they can travel in tandem through the body. Primary vessels and nerves travel longitudinally along the axis of the muscle and give rise to secondary branches that penetrate the different layers of connective tissue at right or oblique angels to the primary vessels and give rise to numerous terminal branches and capillaries. At the endomysium, several capillaries surround each individual muscle fiber, which travel parallel to the muscle fiber axis. Myofibers are also supplied with the axon branch of somatic motor neurons which signal fiber contraction after receiving an impulse.

Skeletal muscle is the most abundant tissue in the body, comprising approximately 40% of the total body weight. Structurally, skeletal muscle is a highly organised tissue, which comprises several bundles of muscle fibers surrounded by connective tissue with different names (epimysium, perimysium, and endomysium) depending on the location [1] (Figure 1). Within the skeletal muscle, there is an abundant supply of blood vessels and nerves, which are essential for maintaining the principal muscle functions such as contraction, oxygen delivery, and waste removal [2].

Skeletal muscle is also a very dynamic tissue with a strong regenerative capacity in response to injury or disease. Regeneration of fibers mainly relies on resident muscle stem cells (MuSCs), also called satellite cells (SCs), which are localised between the basal lamina and the muscle fiber membrane [3]. SCs typically exist in a quiescent state but following injury SCs become activated, proliferate and give rise to myogenic precursor cells, known as myoblasts, which will differentiate into new myotubes and fuse with damaged myofibers, to ultimately mature into functional myofibers [3]. Muscle injuries can occur as a result of a variety of events [4], including direct trauma or mechanical deformation (such as muscle freezing, contusions, lacerations, and strains), indirect causes (such as ischaemia, neurological damage, and underlying chronic diseases), due to genetic disorders (such as muscular dystrophies), or exposure to toxic substances (e.g., cardiotoxins, myotoxins, and barium chloride). The disruption of muscle tissue homeostasis due to injury leads to a time course of events that can be summarised in three sequential but overlapping phases (reviewed in references [5, 6]). During the degeneration/inflammatory phase, the rupture and necrosis of myofibers leads to an activation of a cascade of events that result in the recruitment of leukocytes, initially neutrophils and then proinflammatory CD68+/CD163− (M1) macrophages, involved in the removal of necrotic debris *via* phagocytosis [6]. Between 2 and 4 days after injury, these macrophages undergo a phenotype switch towards an “anti-inflammatory” nonphagocytic CD68−/CD163+/CD206+ (M2) phenotype, that contribute to the termination of inflammation by secretion of anti-inflammatory cytokines such as interleukin-10. This is followed by a regeneration/repair phase, characterised by the activation, proliferation, and differentiation of SCs into myoblasts and then myotubes to replace damaged myofibers. The final phase involves extracellular matrix remodeling and maturation of regenerated myofibers with the recovery of muscle function. In most cases, this system leads to successful muscle repair and regeneration of the injured tissue. For instance, in minor or acute muscle injuries, the activation of the muscle repair and regeneration program often leads to full functional recovery. However, if the duration, frequency, and magnitude of the injury are too great, successful regeneration may not be achieved and instead injured tissue is replaced by connective tissue and fat [7].

Over the past years there has been a growing interest in understanding the cellular and molecular mechanisms underlaying skeletal muscle regeneration with the aim of developing novel therapies. *In vivo* preclinical animal models remain the optimal tools for assessing therapeutic efficacy of novel therapeutic products, which often requires a complete morphologic assessment of tissues for treatment group comparisons. Histopathological scoring is a tool by which semiquantitative data can be obtained from tissues [8]. It usually involves scoring of a lesion's magnitude on an ordinal scale. Several multiparametric, semiquantitative standard scoring systems have been introduced previously for histopathological assessment of tissue lesions in different mouse models of disease [9]. However, fewer semiquantitative scoring systems have been described in the literature for assessing skeletal muscle damage [10–14].

Here, we have developed and validated a novel multiparametric, semiquantitative scoring system which assesses histopathological parameters known to be present in ischaemic muscle tissue [15–17], with excellent inter- and intrarater reliability. This scoring system can be used for assessing the degree of muscle ischaemic damage and for the treatment group comparison in preclinical efficacy studies that use the murine model of hind limb ischaemia (HLI). The use of a reliable and standardised scoring system for a specific disease model will allow more meaningful comparison of results from different studies and laboratories.

## 2. Materials and Methods

### 2.1. Animals

Male 8–10 weeks old BALB/c nude mice were purchased from Envigo (United Kingdom) and were housed in a licensed preclinical facility at Biomedical Science, at the University of Galway, with monitoring and support from qualified animal technicians and a veterinary surgeon. Ethical approval was granted by the Institutional Animal Care Research Ethics Committee. Project authorisation was granted by the Health Products Regulatory Authority in Ireland.

### 2.2. Induction of HLI

**Figure 2:**
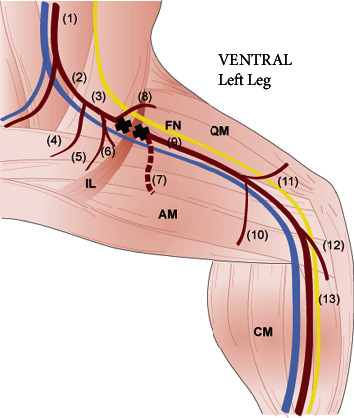
Induction of skeletal muscle ischaemia by ligation and excision of the femoral artery. (1) Aorta artery; (2) common iliac artery; (3) external iliac artery; (4) internal iliac artery; (5) external pudendal artery; (6) caudal epigastric artery; (7) deep femoral artery; (8) iliacofemoral artery; (9) femoral artery; (10) proximal caudal femoral artery; (11) superficial caudal epigastric artery; (12) popliteal artery; (13) saphenous artery; FN = femoral nerve; IL = inguinal ligament; QM = quadricep muscles; AM = adductor muscles; CM = calf muscles.

Unilateral HLI was surgically induced in 8–10 weeks old male BALB/c nude mice. Animals were anesthetised with 75 mg/kg Ketamine and 0.5 mg/kg Dormitor 10 solution injected subcutaneously. The femoral triangle in the left leg was exposed through an incision in the inguinal region and the femoral artery was separated from the femoral vein and nerve by blunt dissection distal to the inguinal ligament. The femoral artery was occluded by placement of sutures at the proximal and distal regions above the proximal caudal femoral artery branch and a cut was made between the ligation sites (Figure 2). The incision was then closed with interrupted sutures. After this, anaesthesia was partially reverted with atipamezole (5 mg/kg). Mice received analgesia (0.05–0.1 mg/kg of buprenorphine 8–12 h for 3 days and as required thereof) and prophylactic antibiotic (0.1 mg/kg of enrofloxacin/Baytril) was also given once postoperatively. Twenty-eight days after ischaemia induction, animals were humanely euthanised, and the body weight of the mice was recorded.

### 2.3. Dissection and Tissue Preparation

For each animal the posterior calf muscles (gastrocnemius, soleus, and plantaris from the fascia) from ischaemic and nonischaemic limbs were carefully dissected as previously reported [18], and their weight was recorded. To adjust for small differences in animal size, calf muscle weights were normalized to the total mouse body weight (calf muscle weight [g]/body weight [g]). Tissue samples were fixed with 10% buffered formalin for 48 h and processed for histological analysis using a tissue processor (Leica ASP3000).

### 2.4. Sectioning and Staining

Tissue cross-sections were taken from the mid-belly of the dissected calf muscles. The calf muscle was cut at mid-belly level and the two pieces were embedded vertically facing down in a paraffin block. Two transverse calf tissue cross-sections of 5 *μ*m in thickness, separated approximately 200 *μ*m from each other, were used for analysis (e.g., total of 4 sections). Tissue sections were deparaffin in xylene and rehydrated through a series of ethanol grade prior to staining with haematoxylin and eosin (H&E) and Mallory's trichrome staining using standard protocols.

### 2.5. Immunohistochemistry

Skeletal muscle fiber membrane was stained with 5 *μ*g/mL fluorescein conjugated wheat germ agglutinin (WGA) (Vector Laboratories) for 10 min and the nuclei of cells were counterstained with DAPI (Fluroshield™ mounting media, Sigma-Aldrich). The whole calf cross-section was scanned at 10x magnification using the EVOS M7000 Slide Scanner Microscope (Invitrogen). Quantitative determination of muscle fiber diameter (minimum Feret's diameter) was performed using Image J software. A total of 1,500–2,000 fibers per muscle were used for analysis.

### 2.6. Scoring System

**Figure 3:**
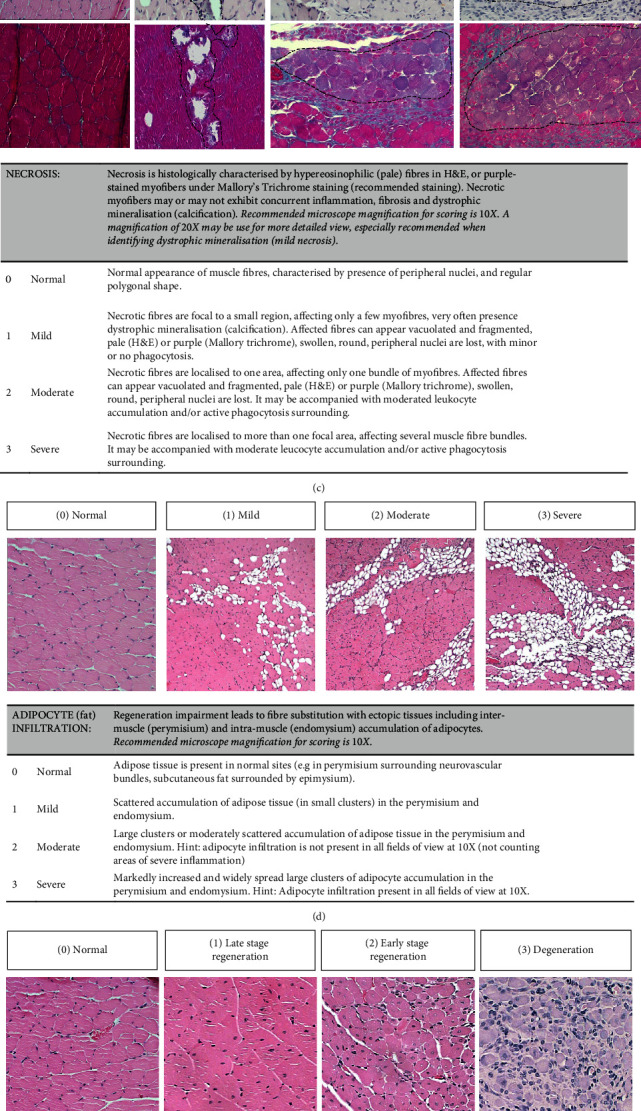
Approach to scoring the histopathological parameters to evaluate ischaemia severity in skeletal muscle. This figure was compiled with guidance from the Thuilliez et al.'s work [35] and from the National Toxicology Program (NTP) Nonneoplastic Lesion Atlas [34].

A total of 70 skeletal muscle samples were scored. These comprised a selection of samples from nonischaemic skeletal muscles (normal) as well as ischaemic skeletal muscles with a wide range of ischaemic damage and regeneration to capture all the proposed levels (as shown in Table 1). Each muscle tissue sample was carefully observed under an Olympus Bx43 bright field microscope by three independent operators. Scoring of each sample was performed by visualising the entire calf muscle cross-sectional area directly under the microscope, not by scoring multiple single frame images taken from the same muscle section (i.e., fields of view). The researchers observed four separated cross-sections for each calf muscle, and a score was given that best represented that particular calf muscle. While in the majority of cases, the same degree of lesion was present across the four cross-sectional areas observed, it was possible that in some cases, the same degree of lesion was not present in all the cross-sections. In this scenario, the major score for that particular lesion was given as demonstrating the highest severity of the pathology present in the muscle. All the microscope magnifications can be used in order to obtain a general view as well as more detailed view of the regions of interest. However, a recommended magnification for scoring each parameter is detailed in Figure 3.

Figure 3 describes the proposed system of evaluation. Here, a detailed description and sample pictures of muscle tissue for each of the categories and levels is included. The histopathological parameters assessed included muscle inflammation, fibrosis, necrosis, adipocyte (fat) infiltration, and muscle fiber degeneration/regeneration. Each parameter was independently scored using an analogue scale of 0 to 3 using a grading sheet (see Supplementary Materials). Additional significant observations were scored +1 if present. Finally, a cumulative ischaemia severity score (cISS) for each muscle sample was obtained by the sum of each individual score. We ensured that a range of different score levels (0 to 3) were present in similar frequencies in each of the categories.

### 2.7. Appraisers

There were 2 male appraisers and 1 female appraiser, who had good experience visualising histological samples prior to performing the scoring. One appraiser is a qualified consultant pathologist (S.O.H), who would have a wide range of experience in scoring histological specimens from many different tissues. The other two appraisers (C.SN and M.C) are scientists who have experience in observing histological specimens in this study area. The same three appraisers were blinded for the study and evaluated each sample independently. In addition, one observer (C.SN) rescored the 70 samples following a 4-month washout period for intraobserver analysis. To ensure that the order of data collection would not influence results, each appraiser evaluated all samples in a random order using a predefined agreement analysis worksheet.

### 2.8. First Consensus Scoring Meeting

Prior to commencing the study, the three appraisers met to discuss the parameters and criteria of this scoring system. The definitions for each morphological parameter were reviewed and any discrepancy or lack of clarity in the scoring definition was addressed. Several sample slides were examined by the three appraisers and a score was given. This was to ensure that appraisers had some level of agreement prior to commencing the study.

### 2.9. Final Conclusion Meeting

A meeting between the three appraisers was arranged at the end of the study to discuss the results. As in some cases appraisers differed in their scores, it was agreed that it was most appropriate to use the median value of the three scores given to each of the individual histological parameters when calculating the cISS. This value was then used to investigate the association between the cISS with a clinical parameter of disease severity such as calf muscle weight and skeletal muscle fiber diameter.

### 2.10. Statistical Analysis

Kendall's coefficient of concordance (*W*) (a nonparametric test of rank-ordering concordance) was used to measure the degree of association of ordinal assessments made by the three appraisers [19]. Kendall *W* accounts for the order of the ratings, for instance, a classification of an observation among raters which differs by 2 points is considered more serious than a classification which only differs by 1 point (e.g., severe (score 3) vs. mild (score 1) is considered worse than severe (score 3) vs. moderate (score 2)). A perfect agreement is indicated by values of 1, while no agreement is indicated by values of 0. Intrarater agreement was assessed similarly. Spearman rank-order correlation and linear regression analysis was performed between muscle weights, minimum Feret's diameter, and the cISS. All statistical analyses were performed in Minitab 19 statistical software. Statistical significance was assigned at *p* value ≤0.05.

## 3. Results

### 3.1. Score Tool Reliability: Interrater and Intrarater Variation for Scoring Histopathological Parameters

Interrater reliability was demonstrated on the basis of a significant level of rank-ordering concordance using Kendall's *W* [20]. For all the histopathological parameters scored, we estimated interrater reliability to be significantly high (Table 2). In most cases, disagreements between the three appraisers were no more than 1 point of difference (e.g., one appraiser gave a score of 3 and another appraiser gave a score of 2). Disagreements among appraisers of >2 points were minimal (Table 2). Complete disagreements (three appraisers completely disagreed in their scores) were also minimal, i.e., agreement among two appraisers was almost 100% in all the cases (Table 2). Necrosis was the parameter with highest % of agreement, but with lower Kendall's *W*. This may be due to having more scores that differed more than 1 point (5/70). Apart from the 5 general histopathological findings assessed, other significant histopathological findings were reported if significant. For instance, abnormal haemorrhage was reported in 2/70 samples. Finally, intrarater reliability was also found to be excellent for all the parameters scored, with Kendall's *W* of 0.94 or greater (Table 3).

### 3.2. Validation of Tissue Pathology: Clinical Measure of Disease Severity

**Figure 4:**
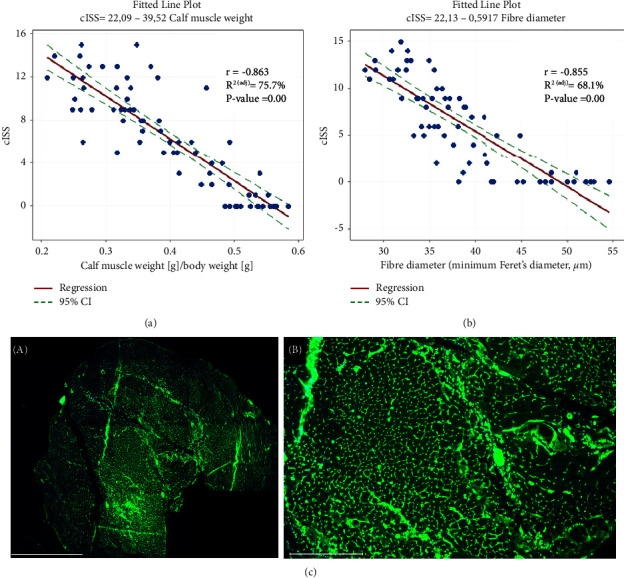
Correlation and linear regression analysis. (a) Cumulative ischaemia severity score (cISS) vs. calf muscle weight ratio (calf muscle weight [g]/body weight [g]). (b) Cumulative ischaemia severity score (cISS) vs. skeletal muscle fiber diameter (minimum Feret's diameter). (c) (A) Representative image of a whole cross-sectional area of skeletal ischaemic calf muscle stained with WGA (green) and DAPI (blue). (B) Representative image at 10x magnification of calf muscle field of view used to quantify skeletal muscle fiber diameter. Scale bar = 1,000 *μ*m (A) and 275 *μ*m (B).

Once appraisers scored all the histopathological parameters, the median value for each parameter was calculated. A cISS per muscle sample was calculated by adding up all the individual scores. The cISS score ranged from 0 (score of 0 in all individual parameters) to 15 (score of 3 in all individual parameters). Additional points (+1) were added to the final score for every other significant histopathological finding (i.e., presence of abnormal haemorrhage). This total score was used to investigate the association between cISS and an objective clinical parameter of disease severity. Calf muscle weight can be a relevant parameter of tissue pathology. We, and others, have observed significant muscle mass loss after ischaemic injury, most likely secondary to muscle necrosis and fibrosis [21, 22]. We hypothesised that the degree of muscle ischaemic damage would be correlated with the level of muscle weight loss and the level of muscle regeneration (e.g., skeletal muscle fiber diameter). Spearman rank-order correlation analysis was performed to investigate the relationship between the cISS score and these two parameters. We found a statistically significant strong negative relationship between cISS scores and calf muscle weights with *r* = −0.863 and 95% CI of −0.920 to −0.772 (*p* ≤ 0.001). A linear regression analysis indicated that there was an indirect linear relationship between these two parameters (*R*^2^adj 75.7%, *p* ≤ 0.001) (Figure 4(a)). In addition, we investigated the relationship between the cISS and the skeletal muscle fiber diameter, as a measure of skeletal muscle regeneration, and found similar results, including a statistically significant string negative relationship with *r* = −0.855 and 95% CI of −0.892, −0.733 *p* ≤ 0.001) and an indirect linear relationship between these two parameters (*R*^2^adj 68.10%, *p* ≤ 0.001) (Figures 4(b) and 4(c)).

## 4. Discussion

Ischaemia in skeletal muscle occurs due to insufficient supply of nutrients and oxygen. In patients with peripheral arterial disease (PAD), ischaemia of distal muscles occurs due to the narrowing or occlusion of peripheral arteries due to the build-up of atherosclerotic plaques [23]. Ischaemic calf muscle in PAD patients is characterised by several histopathological changes such as local inflammation, increased fibrosis and inter- and intramuscle adipocyte content, muscle fiber atrophy, and impaired metabolic function, among others [15, 24]. The most severe manifestation of PAD, namely, critical limb ischaemia (CLI), is characterised by rest pain, nonhealing ulcers, gangrene, tissue loss, and death [23]. In the past years, there has been an increased interest in developing novel therapeutic products aiming to improve tissue perfusion and/or restoration of tissue function in these patients [25]. The mouse model of HLI is considered the most clinically relevant preclinical model of PAD, and especially CLI [26], and has been largely used to assess preclinical efficacy of cell therapy products such as mesenchymal stromal cells (MSCs) [27–31]. In most cases, a complete morphologic assessment of tissue using a range of histological techniques is performed for the treatment group comparisons. However, no standardised tools are used for the assessment of the degree of skeletal muscle damage across all these studies and there exists great variation amongst the histological techniques and quantification methods employed. This may impair interstudy comparability. Semiquantitative histopathology scoring systems have been previously used to obtain semiquantitative data from tissue samples [8]. To our knowledge, there are a small number of studies that have described a semiquantitative scoring system as part of their methodology to assess the level of skeletal muscle damage [10–14]. McCormack et al. described an absolute injury score (i.e., percentage of injury) calculated by dividing the number of injured myocytes by the total myocytes scored within 15 photographed fields (approximately 1,000 fibers per animal) [13]. While this scoring system has the advantage of providing quantitative data (e.g., ratio) from a tissue, it does not provide information about other important histopathological parameters such as the level of inflammation, fibrosis, or others. Erkanli et al. described a histological damage score tool for histological evaluation of tissue sections based on a severity level (0: normal, 1: mild, 2: moderate, 3: severe) of disorganisation and degeneration of muscle fibers and inflammatory cell infiltration [12]. However, no scoring definitions are provided for each category to guide the observer when performing the scoring [12]. This is likely to result in a reduction of intra- and interrater repeatability. Carter et al. described a more comprehensive skeletal muscle histopathology scoring system that scores a lesion's magnitude on an ordinal scale from 0 to 10 [10]. While a score definition is provided for each category to guide the observer during the scoring, each category scores several parameters at once (e.g., the severity of mononuclear cell infiltration, polynuclear cell infiltration, level of fiber necrosis, and presence of haemorrhage). In cases when a tissue has multiple lesions, it is preferable to assign its own appropriate scoring system for each parameter [8]. This approach is more sensitive, and results in higher interrater repeatability. Also, a large number of ordinal scores may cause difficulty or ambiguity during score assignment and is prone to have reduced repeatability [8]. Indeed, Smajović et al. reported a simplified version of the Carter et al. scoring system to include only 4 levels [11]. Finally, Hardy et al. described a morphometric semiquantitative analysis to assess the extent of muscle injury in four different injury models at different timepoints. In this case, a symbol “+” with more or less +'s is given to each morphological parameter depending on the percentage of tissue affected [14].

Here, we have developed and validated a new semiquantitative histopathological scoring tool to assess skeletal muscle damage due to ischaemia with excellent intra- and interrater reliability. We believe our scoring tool has many advantages over the scoring systems described above. We have used the “splitter” approach, where we have assigned a specific score system to different individual parameters (e.g., inflammation, fibrosis, and necrosis), as it is the preferred approach to use when multiple lesions are present in the same tissue [8]. We also used an ordinal scale with a maximum of 4 score levels for each parameter to describe the severity of the lesion, as it has been previously suggested that 4-5 score levels may be optimal for maximising detection and repeatability [8]. In addition, we have provided a comprehensive description of each score level including representative examples to guide the raters and enhance interobserver repeatability, which is unique in the literature when assessing skeletal muscle damage (Figure 3). One of the advantages of this scoring system is that a cISS can be calculated by addition of all the individual scores. This gives an overview of the level of skeletal muscle ischaemic damage when taking in consideration all the histopathological parameters examined in the sample. In Table 1, we have proposed an overall interpretation of the level of muscle ischaemia damage based on the cISS by providing specific cISS intervals for a “normal,” “mild,” “moderate,” and “severe” muscle ischaemic damage. Also, here we can confirm that there is a similar distribution of each of the proposed levels across all the 70 scored samples, which is considered important when designing a new scoring system (Table 1).

**Figure 5:**
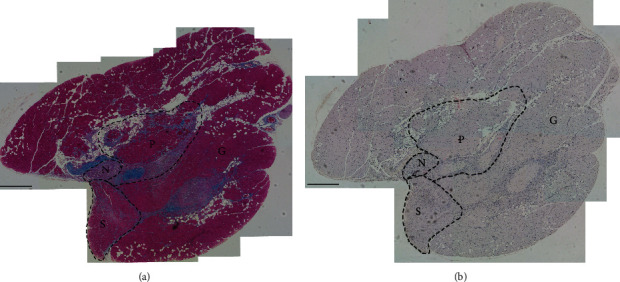
Assembled image of a whole cross-sectional area of ischaemic calf skeletal muscle 28 days after ischaemia. (a) Calf muscle cross-section stained with Mallory's trichrome staining. (b) Calf muscle cross-section stained with H&E. Images were taken with a 10x magnification and assembled to obtain the overall cross-sectional area. Dotted lines highlight approximate edges of plantaris (P), soleus (S), and gastrocnemius (G) muscles. N = tibial nerve. Scale bar is 0.4 mm.

In this study we applied the scoring system to the whole calf muscle cross-section in order to obtain an overall representation of level of ischaemia-induced skeletal muscle damage (Figure 5). The calf muscle consists of three separated muscles, the gastrocnemius, soleus and plantaris muscles, and therefore, this scoring system could also be applied to the three separated muscles independently if required. We have observed some differences in regard to the injury-repair process across the three muscle types, which may be due to the different composition of myofiber types, and metabolic demands, which can affect the overall injury-repair process. For instance, the gastrocnemius muscle is primarily composed of MyHC2B fibers (glycolytic fibers), more abundant in superficial regions, with some MyHC2A and MyC2X in deeper regions. The soleus muscle predominantly presents MyHC1 fibers (slow oxidative fibers) in combination with some MyHC2A fibers; and plantaris muscle is composed primarily of MyHC2B fibers with considerable numbers of MyHC2A and MyHC2X fibers [32].

Overall, we found widespread inflammation and fibrosis across the three muscles 28 days after ischaemia injury (Figure 5). In general, the plantaris muscle was largely affected by ischaemia, with severe inflammation, fibrosis, and necrosis always present. The deeper regions of the gastrocnemius were also largely affected by the ischaemia injury. Fibrosis and inflammation were also observed in the soleus muscle although this muscle seemed to be less affected than the other two muscles (e.g., necrotic clusters of fibers were rarely observed in the soleus). Also, abnormal muscle fat infiltration was rarely observed in the soleus muscle and was most commonly observed in the gastrocnemius muscle. Overall, the soleus muscle seems to recover faster from the injury. In contrast, plantaris and gastrocnemius may still present some histological features, such as fat infiltration or some areas of fibrosis even when inflammation is minimal, and regeneration is ensuing. These findings are in concordance with Charles et al who hypothesised that glycolytic muscles (e.g., gastrocnemius) are more prone to ischaemia-reperfusion-induced injury than oxidative skeletal muscles (e.g., soleus) [33]. Oxidative skeletal muscles are characterised by increased mitochondrial content and enhanced antioxidant defences allowing better protection against ischaemia-reperfusion, while the impaired mitochondrial respiration, increased reactive oxygen species (ROS) production and reduced antioxidant defences found in glycolytic gastrocnemius muscle may be key contributors to the injury. The plantaris muscle is also glycolytic and therefore it is also prone to injury.

### 4.1. How to Use This Tool

We propose that samples should be scored by a minimum of two independent appraisers blinded to the treatments. While adding more appraisers may result in a reduction of the percentage of agreement, the calculation of cISS may become less biased when there are disagreements, as the cISS can be calculated using the median scores among three appraisers. In the cases where two appraisers score the samples, the two appraisers must discuss the disagreements and agree a final score. Furthermore, if the appraisers do not have experience in this study area, we recommend achieving some level of training prior to starting the scoring. The National Toxicology Program (NTP) Nonneoplastic Lesion Atlas is a publicly available web-based resource containing images, terminology, and guidelines for diagnosis of nonneoplastic lesions in rodents [34]. Thuilliez et al. work has compiled a glossary of definitions and pictorial examples of histopathological lesions often observed in skeletal muscle of rodents after intramuscular injection that may guide the researchers when reporting histological findings [35]. Overall, the lowest percentage of agreement for a specific morphological category scored by two selected appraisers was 51% (Table 4). Therefore, we recommend reaching, at least, 50% agreement in scoring each morphological parameter among two selected appraisers prior to commencing the study. This will enhance interrater reliability. When reporting the results, we propose to report both, individual scores and cISS. Average scores (median) of the different experimental groups can be then compared using nonparametric statistical tests. Finally, while this is a simple tool that requires the use of two routine histological stains such as H&E and Mallory trichrome stain (Masson's Trichrome staining is also valid). Nevertheless, we propose that the use of this scoring system can be complemented with other staining and other quantifiable methods that may be relevant to each particular study.

### 4.2. Limitations

We caution that Kendall's *W* does not imply that any particular appraiser is correct or incorrect, simply whether observers agreed or not. We, however, have validated this tool using a clinical measure of disease severity in these mice, such as calf muscle weight. Muscle wasting and weakness is a common symptom in PAD [36]. We and others have observed muscle mass loss after ischaemia in rodent, which is most likely secondary to muscle necrosis and fibrosis and can return to baseline levels with regeneration [21, 22]. Spearman rank-order correlation analysis showed a strong and statistically significant negative relationship between the cISS and calf muscle weight (*r* = −0.863, *p* ≤ 0.001). This convincing finding lends credence and scientific merit to our scoring method, which shows a good representation of the pathology of the tissue. Nevertheless, correlation analysis between skeletal muscle weight and cISS must also be done with caution when using other injury models and/or timepoints. Factors such as adipocyte infiltration, extent of fibrosis or oedema (especially at very early timepoint) [37] may influence muscle weight. In this regard, skeletal muscle fiber size is a reliable and reproducible parameter to indicate muscle fiber regeneration after injury. Here, we have investigated the relationship between cISS and skeletal muscle fiber diameter, and our results showed a strong and statistically significant relationship between these two parameters (*r* = −0.855, *p* ≤ 0.001).

There are other parameters that must be taken in consideration prior to using this tool, including the endpoint of the study at which muscles are scored, and the animal strain. Our *in vivo* study endpoint and assessment has been optimised at 28 days after ischaemia surgery. At this timepoint we have observed significant muscle mass loss compared to the nonischaemic limb, and also muscle gain due to regeneration, which allows the treatment group comparisons (unpublished observations, Sanz-Nogués et al.). However, the study endpoint may differ for other studies. In this regard, one should take caution as the severity of lesions can differ across different endpoints. Moreover, the magnitude and the persistence of the lesion may differ across different types of skeletal muscle injury models. Therefore, the timepoint at which the scoring system is applied may vary across different injury models. In addition to this, it is widely acknowledged that there are differences between inbred strains of mice to surgically induced HLI [38–41]. For instance, C57BL/6 mice showed significantly better collateral artery formation and limb perfusion, and less tissue damage than BALB/c mice in response to HLI [39–41]. BALB/c mice have significantly lower expression of vascular endothelial growth factor A (VEGF-A), poor collateral artery formation, reduced limb perfusion, and impaired recovery [39–41], as well as significantly greater myofiber atrophy, greater apoptosis, and attenuated myogenic regulatory gene expression than C57BL/6 mice [38]. In cases when different animal strains and/or study endpoints are utilised, we recommend first evaluating whether the range of lesions present in the samples can be assessed using the lesion severity proposed in this scoring system for each parameter evaluated.

## 5. Conclusion

Here, we have developed and validated a novel multiparametric semiquantitative scoring system that can be used to evaluate the level of ischaemia-induced muscle damage with excellent inter- and intrarater reliability. We propose that this tool can be used for treatment comparisons in preclinical animal models such as the HLI mouse model. Nevertheless, we anticipate that the use of this tool can be extended for assessing muscle damage due to other injuries, as the process of muscle repair and regeneration, as well as the histopathological features evaluated here, have been found to be quite similar in other skeletal muscle injury models widely employed to study regeneration, including cardiotoxin, freeze injury, barium chloride, and notexin injury [14, 22, 37]. As publications on validated multiparametric semiquantitative scoring system for muscle injury are rare, we believe that this article is an important contribution to the very limited database of published scoring systems.

## Figures and Tables

**Table 1:** Interpretation of the overall level of muscle ischaemia damage samples based on cISS.

cISS	Levels of muscle ischaemia damage	Distribution of levels in scored samples (%)
0-1	Normal	24.3
2–6	Mild	27.1
7–10	Moderate	24.3
11–15+	Severe	24.3

cISS: cumulative ischaemia severity score.

**Table 2:** Kendall's *W* analysis reflecting interrater reliability assessment of histopathological features between three appraisers.

Histopathological parameters	% complete agreements (95% CI)^*∗*^	% agreement between 2 appraisals (95% CI)	% disagreement >2 scores (95% CI)	Kendall's *W*^*∗*^	P value^*∗*^
Inflammation	44.3 (32.4, 56.6)	97.1 (90.1, 99.7)	5.7 (1.6, 14.0)	0.923	≤0.001
Fibrosis	51.4 (39.2, 63.6)	100 (94.9, 100)	0.0 (0.0, 5.1)	0.942	≤0.001
Necrosis	58.6 (46.2, 70.2)	98.6 (92.3, 100)	7.1 (2.3, 15.9)	0.771	≤0.001
Degeneration/regeneration	47.1 (35.1, 59.5)	94.3 (86.0, 98.4)	7.1 (2.3, 15.9)	0.863	≤0.001
Adipocyte infiltration	41.4 (29.8, 53.83)	98.6 (92.3, 100)	5.7 (1.6, 14.0)	0.914	≤0.001

CI: confidence interval; *W*: Kendall's coefficient of concordance; ^*∗*^all appraisers' assessments agree with each other. ^*∗*^*p* values were calculated based on Kendall's *W* scores from complete agreements.

**Table 3:** Kendall's *W* analysis reflecting intrarater reliability assessment of histopathological features by one appraiser after a 4-month washout period.

Histopathological parameters	% agreement (95% CI)	Kendall's *W*	P value
Inflammation	82.7 (71.9, 90.8)	0.978	≤0.001
Fibrosis	85.7 (75.3, 92.9)	0.977	≤0.001
Necrosis	90.0 (80.5, 95.9)	0.951	≤0.001
Degeneration/regeneration	77.1 (65.5, 86.3)	0.943	≤0.001
Adipocyte infiltration	88.6 (78.7, 94.9)	0.977	≤0.001

CI: confidence interval; *W*: Kendall's coefficient of concordance.

**Table 4:** Percentage of agreement among two selected appraisers.

Rater comparisons	Rater 1 vs 2	Rater 1 vs 3	Rater 2 vs 3
Inflammation	65.7 (53.4, 76.6)	71.4 (59.4, 81.6)	51.4 (39.2, 63.6)
Fibrosis	70.0 (57.9, 80.0)	71.4 (59.4, 81.6)	58.6 (46.2, 20.2)
Necrosis	74.3 (62.4, 83.9)	55.7 (64.0, 85.2)	62.9 (51.5, 74.2)
Degeneration/regeneration	65.7 (53.4, 76.6)	65.7 (53.4, 76.6)	57.2 (44.7, 68.9)
Fat infiltration	58.6 (46.2, 20.2)	70.0 (57.9, 80.0)	54.3 (41.9, 66.3)

Results are expressed as % agreement (95% confidence interval).

## Data Availability

The authors declare that the data supporting the findings of this study are available within the article and its supplementary information files. The raw data generated and analysed during the current study are available in Supplementary Materials.

## Supplementary Materials

Supplementary Materials Supplementary Figure 1: a grading sheet template. This template can be used by the researchers when rating histological samples using this semiquantitative scoring system. Supplementary Figure 2: the interrater and intrarater scoring raw data.
