# Supplementary material for: Development and Validation of a Multiparametric Semiquantitative Scoring System for the Histopathological Assessment of Ischaemia Severity in Skeletal Muscle
Source: J Tissue Eng Regen Med. 2023 Mar 16;2023:5592455. doi: 10.1155/2023/5592455 (PMC11918935; doi:10.1155/2023/5592455)
Supplement: Supplementary Materials — Supplementary Figure 1: a grading sheet template. This template can be used by the researchers when rating histological samples using this semiquantitative scoring system. Supplementary Figure 2: the interrater and intrarater scoring raw data. [file 5592455.f1.zip › Additional File 1.docx]

**Additional file 1:** Rater grading sheet template.

| **SKELETAL MUSCLE GRADING SHEET** | | | |
| --- | --- | --- | --- |
| Rater Name: |  | Date: |  |
| Animal #: |  | Slide #: |  |

| **PARAMETER** | | **SCORE OPTIONS** | | **SCORE HERE** | **COMMENTS** |
| --- | --- | --- | --- | --- | --- |
| **INFLAMMATION** | 0 | | Normal |  |  |
|  | 1 | | Mild |  |  |
|  | 2 | | Moderate |  |  |
|  | 3 | | Severe |  |  |
| **FIBROSIS** | 0 | | Normal |  |  |
|  | 1 | | Mild |  |  |
|  | 2 | | Moderate |  |  |
|  | 3 | | Severe |  |  |
| **NECROSIS** | 0 | | Normal |  |  |
|  | 1 | | Mild |  |  |
|  | 2 | | Moderate |  |  |
|  | 3 | | Severe |  |  |
| **FIBRE DEGENERATION / REGENERATION** | 0 | | Normal |  |  |
|  | 1 | | Late-stage regeneration |  |  |
|  | 2 | | Early-stage regeneration |  |  |
|  | 3 | | Fibre degeneration |  |  |
| **ADIPOCYTE ACCUMULATION** | 0 | | Normal |  |  |
|  | 1 | | Mild |  |  |
|  | 2 | | Moderate |  |  |
|  | 3 | | Severe |  |  |
| **MUSCLE HAEMORRHAGE** | 0 | | Absence |  |  |
|  | 1 | | Presence |  |  |
| **OTHER** | 0 | | Absence |  |  |
|  | 1 | | Presence |  |  |
