# Supplementary material for: Development and Validation of a Multiparametric Semiquantitative Scoring System for the Histopathological Assessment of Ischaemia Severity in Skeletal Muscle
Source: J Tissue Eng Regen Med. 2023 Mar 16;2023:5592455. doi: 10.1155/2023/5592455 (PMC11918935; doi:10.1155/2023/5592455)
Supplement: Supplementary Materials — Supplementary Figure 1: a grading sheet template. This template can be used by the researchers when rating histological samples using this semiquantitative scoring system. Supplementary Figure 2: the interrater and intrarater scoring raw data. [file 5592455.f1.zip › Additional File 2.docx]

**Additional File 2:**

**Inter-rater scoring raw data.**

|  | **Inflammation** | | | | **Fibrosis** | | | | **Necrosis** | | | | **De-/Regeneration** | | | | **Fat accumulation** | | | | **Haemorrhage** | | | |  |
| --- | --- | --- | --- | --- | --- | --- | --- | --- | --- | --- | --- | --- | --- | --- | --- | --- | --- | --- | --- | --- | --- | --- | --- | --- | --- |
| **ID** | **R.1** | **R.2** | **R.3** | **Score** | **R.1** | **R.2** | **R.3** | **Score** | **R.1** | **R.2** | **R.3** | **Score** | **R.1** | **R.2** | **R.3** | **Score** | **R.1** | **R.2** | **R.3** | **Score** | **R.1** | **R.2** | **R.3** | **Score** | **cISS** |
| **1** | 3 | 1 | 3 | 3 | 3 | 2 | 3 | 3 | 2 | 2 | 2 | 2 | 2 | 3 | 3 | 3 | 2 | 1 | 1 | 1 | 1 | 1 | 1 | 1 | 13 |
| **2** | 0 | 0 | 0 | 0 | 0 | 0 | 0 | 0 | 0 | 0 | 0 | 0 | 0 | 0 | 0 | 0 | 0 | 0 | 0 | 0 | 0 | 0 | 0 | 0 | 0 |
| **3** | 2 | 1 | 2 | 2 | 2 | 2 | 3 | 2 | 0 | 0 | 0 | 0 | 3 | 2 | 3 | 3 | 3 | 2 | 3 | 3 | 0 | 0 | 0 | 0 | 10 |
| **4** | 3 | 3 | 3 | 3 | 3 | 3 | 3 | 3 | 1 | 3 | 1 | 1 | 3 | 3 | 3 | 3 | 3 | 2 | 2 | 2 | 0 | 0 | 0 | 0 | 12 |
| **5** | 1 | 1 | 2 | 1 | 1 | 1 | 1 | 1 | 0 | 0 | 0 | 0 | 2 | 2 | 2 | 2 | 1 | 1 | 1 | 1 | 0 | 0 | 0 | 0 | 5 |
| **6** | 1 | 0 | 0 | 0 | 1 | 0 | 0 | 0 | 1 | 1 | 0 | 1 | 1 | 1 | 0 | 1 | 1 | 0 | 0 | 0 | 0 | 0 | 0 | 0 | 2 |
| **7** | 3 | 1 | 1 | 1 | 3 | 2 | 3 | 3 | 0 | 1 | 0 | 0 | 3 | 2 | 3 | 3 | 3 | 2 | 2 | 2 | 0 | 0 | 0 | 0 | 9 |
| **8** | 3 | 2 | 3 | 3 | 3 | 2 | 3 | 3 | 2 | 0 | 0 | 0 | 3 | 2 | 3 | 3 | 3 | 2 | 3 | 3 | 0 | 0 | 0 | 0 | 12 |
| **9** | 2 | 1 | 1 | 1 | 2 | 1 | 2 | 2 | 1 | 1 | 0 | 1 | 3 | 2 | 2 | 2 | 3 | 1 | 3 | 3 | 0 | 0 | 0 | 0 | 9 |
| **10** | 3 | 3 | 3 | 3 | 3 | 3 | 3 | 3 | 3 | 2 | 3 | 3 | 3 | 3 | 3 | 3 | 2 | 1 | 1 | 1 | 0 | 0 | 0 | 0 | 13 |
| **11** | 0 | 0 | 0 | 0 | 0 | 0 | 0 | 0 | 0 | 0 | 0 | 0 | 0 | 0 | 0 | 0 | 0 | 0 | 0 | 0 | 0 | 0 | 0 | 0 | 0 |
| **12** | 3 | 2 | 3 | 3 | 3 | 2 | 3 | 3 | 3 | 2 | 3 | 3 | 3 | 2 | 3 | 3 | 3 | 2 | 2 | 2 | 0 | 0 | 0 | 0 | 14 |
| **13** | 0 | 0 | 0 | 0 | 0 | 0 | 0 | 0 | 0 | 0 | 0 | 0 | 0 | 0 | 0 | 0 | 0 | 0 | 0 | 0 | 0 | 0 | 0 | 0 | 0 |
| **14** | 2 | 1 | 2 | 2 | 2 | 1 | 2 | 2 | 1 | 1 | 0 | 1 | 1 | 1 | 2 | 1 | 2 | 1 | 2 | 2 | 0 | 0 | 0 | 0 | 8 |
| **15** | 2 | 1 | 1 | 1 | 2 | 1 | 1 | 1 | 0 | 1 | 0 | 0 | 1 | 1 | 2 | 1 | 2 | 1 | 2 | 2 | 0 | 0 | 0 | 0 | 5 |
| **16** | 1 | 1 | 1 | 1 | 2 | 1 | 1 | 1 | 1 | 1 | 0 | 1 | 1 | 1 | 2 | 1 | 2 | 1 | 2 | 2 | 0 | 0 | 0 | 0 | 6 |
| **17** | 0 | 0 | 0 | 0 | 0 | 0 | 0 | 0 | 0 | 0 | 0 | 0 | 1 | 1 | 1 | 1 | 0 | 0 | 0 | 0 | 0 | 0 | 0 | 0 | 1 |
| **18** | 0 | 0 | 0 | 0 | 0 | 0 | 0 | 0 | 0 | 0 | 0 | 0 | 0 | 0 | 0 | 0 | 0 | 0 | 0 | 0 | 0 | 0 | 0 | 0 | 0 |
| **19** | 3 | 2 | 3 | 3 | 2 | 2 | 3 | 2 | 2 | 1 | 0 | 1 | 3 | 1 | 3 | 3 | 2 | 1 | 2 | 2 | 0 | 0 | 0 | 0 | 11 |
| **20** | 1 | 1 | 2 | 1 | 2 | 1 | 2 | 2 | 1 | 1 | 0 | 1 | 2 | 2 | 2 | 2 | 3 | 2 | 3 | 3 | 0 | 0 | 0 | 0 | 9 |
| **21** | 2 | 1 | 2 | 2 | 2 | 1 | 2 | 2 | 0 | 1 | 0 | 0 | 2 | 2 | 2 | 2 | 3 | 2 | 3 | 3 | 0 | 0 | 0 | 0 | 9 |
| **22** | 3 | 1 | 3 | 3 | 3 | 2 | 3 | 3 | 2 | 1 | 1 | 1 | 3 | 2 | 3 | 3 | 3 | 2 | 3 | 3 | 0 | 0 | 0 | 0 | 13 |
| **23** | 0 | 0 | 0 | 0 | 0 | 0 | 0 | 0 | 0 | 0 | 0 | 0 | 0 | 0 | 0 | 0 | 0 | 0 | 1 | 0 | 0 | 0 | 0 | 0 | 0 |
| **24** | 2 | 2 | 2 | 2 | 1 | 1 | 2 | 1 | 0 | 0 | 0 | 0 | 2 | 0 | 1 | 1 | 1 | 1 | 1 | 1 | 0 | 0 | 0 | 0 | 5 |
| **25** | 1 | 1 | 1 | 1 | 1 | 1 | 1 | 1 | 1 | 1 | 1 | 1 | 2 | 2 | 2 | 2 | 1 | 2 | 2 | 2 | 0 | 0 | 1 | 0 | 7 |
| **26** | 0 | 0 | 0 | 0 | 0 | 0 | 0 | 0 | 0 | 0 | 0 | 0 | 0 | 0 | 0 | 0 | 0 | 0 | 0 | 0 | 0 | 0 | 0 | 0 | 0 |
| **27** | 0 | 0 | 1 | 0 | 1 | 0 | 0 | 0 | 0 | 0 | 0 | 0 | 2 | 2 | 2 | 2 | 0 | 0 | 0 | 0 | 0 | 0 | 0 | 0 | 2 |
| **28** | 3 | 2 | 3 | 3 | 3 | 2 | 3 | 3 | 0 | 1 | 0 | 0 | 3 | 3 | 3 | 3 | 3 | 2 | 3 | 3 | 0 | 0 | 0 | 0 | 12 |
| **29** | 1 | 1 | 1 | 1 | 1 | 1 | 1 | 1 | 1 | 1 | 0 | 1 | 2 | 2 | 1 | 2 | 1 | 1 | 3 | 1 | 0 | 0 | 0 | 0 | 6 |
| **30** | 2 | 1 | 2 | 2 | 2 | 2 | 2 | 2 | 0 | 1 | 1 | 1 | 2 | 2 | 2 | 2 | 1 | 1 | 1 | 1 | 0 | 0 | 0 | 0 | 8 |
| **31** | 0 | 0 | 0 | 0 | 0 | 0 | 0 | 0 | 0 | 0 | 0 | 0 | 0 | 2 | 1 | 1 | 0 | 0 | 0 | 0 | 0 | 0 | 0 | 0 | 1 |
| **32** | 1 | 1 | 1 | 1 | 1 | 1 | 1 | 1 | 1 | 1 | 0 | 1 | 1 | 2 | 1 | 1 | 1 | 1 | 1 | 1 | 0 | 0 | 0 | 0 | 5 |
| **33** | 2 | 2 | 2 | 2 | 2 | 2 | 2 | 2 | 0 | 2 | 0 | 0 | 2 | 3 | 3 | 3 | 3 | 2 | 2 | 2 | 0 | 0 | 0 | 0 | 9 |
| **34** | 0 | 0 | 0 | 0 | 0 | 0 | 0 | 0 | 0 | 0 | 0 | 0 | 1 | 2 | 0 | 1 | 0 | 0 | 0 | 0 | 0 | 0 | 0 | 0 | 1 |
| **35** | 2 | 1 | 2 | 2 | 2 | 2 | 2 | 2 | 0 | 2 | 0 | 0 | 2 | 2 | 3 | 2 | 3 | 3 | 3 | 3 | 0 | 0 | 0 | 0 | 9 |
| **36** | 2 | 2 | 1 | 2 | 2 | 2 | 1 | 2 | 2 | 2 | 2 | 2 | 2 | 3 | 2 | 2 | 3 | 3 | 3 | 3 | 0 | 0 | 0 | 0 | 11 |
| **37** | 1 | 1 | 2 | 1 | 1 | 1 | 2 | 1 | 1 | 1 | 1 | 1 | 2 | 2 | 2 | 2 | 1 | 1 | 2 | 1 | 0 | 0 | 0 | 0 | 6 |
| **38** | 2 | 1 | 3 | 2 | 2 | 2 | 3 | 2 | 1 | 1 | 0 | 1 | 2 | 2 | 3 | 2 | 3 | 2 | 3 | 3 | 0 | 0 | 0 | 0 | 10 |
| **39** | 0 | 0 | 0 | 0 | 0 | 0 | 0 | 0 | 0 | 0 | 0 | 0 | 1 | 2 | 0 | 1 | 1 | 1 | 1 | 1 | 0 | 0 | 0 | 0 | 2 |
| **40** | 0 | 0 | 1 | 0 | 1 | 1 | 0 | 1 | 0 | 0 | 0 | 0 | 1 | 2 | 2 | 2 | 2 | 2 | 3 | 2 | 0 | 0 | 0 | 0 | 5 |
| **41** | 0 | 0 | 1 | 0 | 0 | 0 | 0 | 0 | 0 | 0 | 0 | 0 | 0 | 0 | 1 | 0 | 0 | 0 | 0 | 0 | 0 | 0 | 0 | 0 | 0 |
| **42** | 3 | 2 | 3 | 3 | 3 | 2 | 3 | 3 | 3 | 2 | 3 | 3 | 3 | 3 | 3 | 3 | 3 | 2 | 3 | 3 | 0 | 0 | 0 | 0 | 15 |
| **43** | 2 | 1 | 2 | 2 | 2 | 1 | 2 | 2 | 1 | 1 | 0 | 1 | 2 | 2 | 3 | 2 | 2 | 1 | 2 | 2 | 0 | 0 | 0 | 0 | 9 |
| **44** | 3 | 3 | 3 | 3 | 3 | 3 | 3 | 3 | 3 | 3 | 3 | 3 | 3 | 3 | 3 | 3 | 3 | 3 | 3 | 3 | 0 | 0 | 0 | 0 | 15 |
| **45** | 0 | 0 | 0 | 0 | 0 | 0 | 0 | 0 | 0 | 0 | 0 | 0 | 0 | 0 | 0 | 0 | 0 | 0 | 0 | 0 | 0 | 0 | 0 | 0 | 0 |
| **46** | 1 | 1 | 1 | 1 | 1 | 1 | 1 | 1 | 1 | 1 | 1 | 1 | 2 | 2 | 3 | 2 | 2 | 1 | 3 | 1 | 1 | 0 | 1 | 1 | 7 |
| **47** | 0 | 0 | 0 | 0 | 0 | 0 | 1 | 0 | 0 | 0 | 0 | 0 | 0 | 0 | 0 | 0 | 0 | 0 | 0 | 0 | 0 | 0 | 0 | 0 | 0 |
| **48** | 0 | 0 | 0 | 0 | 0 | 0 | 0 | 0 | 0 | 0 | 0 | 0 | 0 | 0 | 0 | 0 | 0 | 0 | 0 | 0 | 0 | 0 | 0 | 0 | 0 |
| **49** | 1 | 0 | 1 | 1 | 2 | 1 | 2 | 2 | 1 | 0 | 1 | 1 | 2 | 2 | 2 | 2 | 2 | 1 | 2 | 2 | 0 | 0 | 0 | 0 | 8 |
| **50** | 3 | 2 | 3 | 3 | 3 | 2 | 3 | 3 | 3 | 2 | 3 | 3 | 3 | 2 | 3 | 3 | 2 | 2 | 3 | 2 | 0 | 0 | 0 | 0 | 14 |
| **51** | 0 | 0 | 0 | 0 | 0 | 0 | 0 | 0 | 0 | 0 | 0 | 0 | 0 | 0 | 0 | 0 | 0 | 0 | 0 | 0 | 0 | 0 | 0 | 0 | 0 |
| **52** | 0 | 0 | 1 | 0 | 1 | 1 | 1 | 1 | 0 | 0 | 0 | 0 | 1 | 2 | 2 | 2 | 1 | 1 | 2 | 1 | 0 | 0 | 0 | 0 | 4 |
| **53** | 2 | 2 | 3 | 2 | 2 | 2 | 3 | 2 | 0 | 0 | 0 | 0 | 2 | 2 | 3 | 2 | 2 | 1 | 2 | 2 | 0 | 0 | 0 | 0 | 8 |
| **54** | 3 | 3 | 3 | 3 | 3 | 3 | 3 | 3 | 0 | 0 | 1 | 0 | 3 | 3 | 3 | 3 | 3 | 3 | 3 | 3 | 0 | 0 | 0 | 0 | 12 |
| **55** | 2 | 1 | 2 | 2 | 2 | 2 | 3 | 2 | 0 | 0 | 0 | 0 | 2 | 2 | 3 | 2 | 2 | 1 | 2 | 2 | 0 | 0 | 0 | 0 | 8 |
| **56** | 0 | 0 | 0 | 0 | 0 | 0 | 0 | 0 | 0 | 0 | 0 | 0 | 2 | 2 | 2 | 2 | 1 | 1 | 1 | 1 | 0 | 0 | 0 | 0 | 3 |
| **57** | 0 | 0 | 0 | 0 | 0 | 0 | 0 | 0 | 0 | 0 | 0 | 0 | 0 | 0 | 0 | 0 | 0 | 0 | 0 | 0 | 0 | 0 | 0 | 0 | 0 |
| **58** | 2 | 1 | 3 | 2 | 2 | 2 | 3 | 2 | 1 | 0 | 1 | 1 | 2 | 2 | 3 | 2 | 2 | 2 | 2 | 2 | 0 | 0 | 0 | 0 | 9 |
| **59** | 3 | 2 | 3 | 3 | 3 | 3 | 3 | 3 | 2 | 2 | 2 | 2 | 3 | 3 | 3 | 3 | 1 | 1 | 2 | 1 | 0 | 0 | 0 | 0 | 12 |
| **60** | 1 | 1 | 1 | 1 | 1 | 1 | 1 | 1 | 0 | 0 | 0 | 0 | 2 | 1 | 2 | 2 | 2 | 2 | 3 | 2 | 0 | 0 | 0 | 0 | 6 |
| **61** | 0 | 0 | 1 | 0 | 1 | 1 | 0 | 1 | 0 | 0 | 0 | 0 | 2 | 1 | 1 | 1 | 1 | 0 | 1 | 1 | 0 | 0 | 0 | 0 | 3 |
| **62** | 3 | 3 | 3 | 3 | 3 | 3 | 3 | 3 | 3 | 3 | 3 | 3 | 3 | 3 | 3 | 3 | 1 | 1 | 2 | 1 | 0 | 0 | 0 | 0 | 13 |
| **63** | 0 | 0 | 0 | 0 | 0 | 0 | 0 | 0 | 0 | 0 | 0 | 0 | 0 | 0 | 0 | 0 | 0 | 0 | 0 | 0 | 0 | 0 | 0 | 0 | 0 |
| **64** | 3 | 3 | 3 | 3 | 3 | 2 | 3 | 3 | 0 | 1 | 0 | 0 | 3 | 2 | 3 | 3 | 2 | 1 | 2 | 2 | 0 | 0 | 0 | 0 | 11 |
| **65** | 1 | 1 | 2 | 1 | 1 | 1 | 2 | 1 | 0 | 0 | 0 | 0 | 1 | 1 | 1 | 1 | 1 | 1 | 3 | 1 | 0 | 0 | 0 | 0 | 4 |
| **66** | 3 | 2 | 3 | 3 | 3 | 3 | 3 | 3 | 0 | 0 | 0 | 0 | 3 | 3 | 3 | 3 | 2 | 1 | 2 | 2 | 0 | 0 | 0 | 0 | 11 |
| **67** | 0 | 0 | 1 | 0 | 0 | 0 | 0 | 0 | 0 | 0 | 0 | 0 | 0 | 0 | 0 | 0 | 0 | 0 | 0 | 0 | 0 | 0 | 0 | 0 | 0 |
| **68** | 1 | 1 | 2 | 1 | 1 | 2 | 2 | 2 | 0 | 0 | 0 | 0 | 2 | 1 | 2 | 2 | 1 | 1 | 2 | 1 | 0 | 0 | 0 | 0 | 6 |
| **69** | 0 | 1 | 1 | 1 | 0 | 0 | 0 | 0 | 0 | 0 | 0 | 0 | 2 | 1 | 1 | 1 | 1 | 1 | 2 | 1 | 0 | 0 | 0 | 0 | 3 |
| **70** | 1 | 1 | 2 | 1 | 1 | 2 | 2 | 2 | 0 | 0 | 1 | 0 | 2 | 1 | 2 | 2 | 1 | 1 | 1 | 1 | 0 | 0 | 0 | 0 | 6 |

*R.1 = rater 1*

*R.2 = rater 2*

*R.3 = rater 3*

**Intra-rater scoring raw data.**

|  | **Inflammation** | |  | **Fibrosis** | |  | **Necrosis** | |  | **De-/Regeneration** | |  | **Fat accumulation** | |  | **Haemorrhage** | |
| --- | --- | --- | --- | --- | --- | --- | --- | --- | --- | --- | --- | --- | --- | --- | --- | --- | --- |
| **ID** | **1^st^ Time** | **2^nd^ Time** |  | **1^st^ Time** | **2^nd^ Time** |  | **1^st^ Time** | **2^nd^ Time** |  | **1^st^ Time** | **2^nd^ Time** |  | **1^st^ Time** | **2^nd^ Time** |  | **1^st^ Time** | **2^nd^ Time** |
| **1** | 3 | 3 |  | 3 | 3 |  | 2 | 2 |  | 2 | 3 |  | 2 | 2 |  | 1 | 1 |
| **2** | 0 | 0 |  | 0 | 0 |  | 0 | 0 |  | 0 | 0 |  | 0 | 0 |  | 0 | 0 |
| **3** | 2 | 3 |  | 2 | 3 |  | 0 | 0 |  | 3 | 3 |  | 3 | 3 |  | 0 | 0 |
| **4** | 3 | 3 |  | 3 | 3 |  | 2 | 1 |  | 3 | 3 |  | 3 | 3 |  | 0 | 0 |
| **5** | 1 | 2 |  | 1 | 2 |  | 0 | 0 |  | 2 | 2 |  | 1 | 1 |  | 0 | 0 |
| **6** | 1 | 1 |  | 1 | 1 |  | 1 | 1 |  | 1 | 1 |  | 1 | 1 |  | 0 | 0 |
| **7** | 3 | 3 |  | 3 | 3 |  | 0 | 0 |  | 3 | 3 |  | 3 | 3 |  | 0 | 0 |
| **8** | 3 | 3 |  | 3 | 3 |  | 2 | 2 |  | 3 | 3 |  | 3 | 3 |  | 0 | 0 |
| **9** | 2 | 2 |  | 2 | 2 |  | 1 | 1 |  | 3 | 2 |  | 3 | 2 |  | 0 | 0 |
| **10** | 3 | 3 |  | 3 | 3 |  | 3 | 3 |  | 3 | 3 |  | 2 | 2 |  | 0 | 0 |
| **11** | 0 | 0 |  | 0 | 0 |  | 0 | 0 |  | 0 | 0 |  | 0 | 0 |  | 0 | 0 |
| **12** | 3 | 3 |  | 3 | 3 |  | 3 | 3 |  | 3 | 3 |  | 3 | 3 |  | 0 | 0 |
| **13** | 0 | 0 |  | 0 | 0 |  | 0 | 0 |  | 0 | 0 |  | 0 | 0 |  | 0 | 0 |
| **14** | 2 | 2 |  | 2 | 2 |  | 1 | 1 |  | 1 | 2 |  | 2 | 2 |  | 0 | 0 |
| **15** | 2 | 2 |  | 2 | 2 |  | 0 | 0 |  | 1 | 1 |  | 2 | 2 |  | 0 | 0 |
| **16** | 1 | 2 |  | 2 | 2 |  | 1 | 1 |  | 1 | 2 |  | 2 | 2 |  | 0 | 0 |
| **17** | 0 | 0 |  | 0 | 0 |  | 0 | 0 |  | 1 | 1 |  | 0 | 0 |  | 0 | 0 |
| **18** | 0 | 0 |  | 0 | 0 |  | 0 | 0 |  | 0 | 0 |  | 0 | 0 |  | 0 | 0 |
| **19** | 3 | 3 |  | 2 | 3 |  | 2 | 2 |  | 3 | 3 |  | 2 | 2 |  | 0 | 0 |
| **20** | 1 | 2 |  | 2 | 2 |  | 1 | 1 |  | 2 | 2 |  | 3 | 3 |  | 0 | 0 |
| **21** | 2 | 2 |  | 2 | 2 |  | 0 | 0 |  | 2 | 2 |  | 3 | 3 |  | 0 | 0 |
| **22** | 3 | 3 |  | 3 | 3 |  | 2 | 2 |  | 3 | 3 |  | 3 | 3 |  | 0 | 0 |
| **23** | 0 | 0 |  | 0 | 1 |  | 0 | 0 |  | 0 | 1 |  | 0 | 1 |  | 0 | 0 |
| **24** | 2 | 2 |  | 1 | 2 |  | 0 | 0 |  | 2 | 2 |  | 1 | 1 |  | 0 | 0 |
| **25** | 1 | 1 |  | 1 | 1 |  | 1 | 1 |  | 2 | 2 |  | 1 | 1 |  | 0 | 0 |
| **26** | 0 | 0 |  | 0 | 0 |  | 0 | 0 |  | 0 | 0 |  | 0 | 0 |  | 0 | 0 |
| **27** | 0 | 1 |  | 1 | 1 |  | 0 | 0 |  | 2 | 2 |  | 0 | 0 |  | 0 | 0 |
| **28** | 3 | 3 |  | 3 | 3 |  | 0 | 0 |  | 3 | 3 |  | 3 | 3 |  | 0 | 0 |
| **29** | 1 | 1 |  | 1 | 1 |  | 1 | 1 |  | 2 | 2 |  | 1 | 2 |  | 0 | 0 |
| **30** | 2 | 3 |  | 2 | 2 |  | 0 | 0 |  | 2 | 1 |  | 1 | 1 |  | 0 | 0 |
| **31** | 0 | 0 |  | 0 | 0 |  | 0 | 0 |  | 0 | 0 |  | 0 | 0 |  | 0 | 0 |
| **32** | 1 | 1 |  | 1 | 1 |  | 1 | 1 |  | 1 | 1 |  | 1 | 1 |  | 0 | 0 |
| **33** | 2 | 2 |  | 2 | 2 |  | 0 | 0 |  | 2 | 2 |  | 3 | 2 |  | 0 | 0 |
| **34** | 0 | 0 |  | 0 | 0 |  | 0 | 0 |  | 1 | 1 |  | 0 | 0 |  | 0 | 0 |
| **35** | 2 | 2 |  | 2 | 2 |  | 0 | 0 |  | 2 | 3 |  | 3 | 3 |  | 0 | 0 |
| **36** | 2 | 2 |  | 2 | 2 |  | 2 | 2 |  | 2 | 2 |  | 3 | 3 |  | 0 | 0 |
| **37** | 1 | 2 |  | 1 | 2 |  | 1 | 1 |  | 2 | 2 |  | 1 | 1 |  | 0 | 0 |
| **38** | 2 | 2 |  | 2 | 2 |  | 1 | 0 |  | 2 | 2 |  | 3 | 3 |  | 1 | 0 |
| **39** | 0 | 0 |  | 0 | 0 |  | 0 | 0 |  | 1 | 0 |  | 1 | 0 |  | 0 | 0 |
| **40** | 0 | 1 |  | 1 | 1 |  | 0 | 0 |  | 1 | 2 |  | 2 | 2 |  | 0 | 0 |
| **41** | 0 | 0 |  | 0 | 0 |  | 0 | 0 |  | 0 | 0 |  | 0 | 0 |  | 0 | 0 |
| **42** | 3 | 3 |  | 3 | 3 |  | 3 | 2 |  | 3 | 3 |  | 3 | 3 |  | 0 | 0 |
| **43** | 2 | 2 |  | 2 | 2 |  | 1 | 1 |  | 2 | 2 |  | 2 | 2 |  | 0 | 0 |
| **44** | 3 | 3 |  | 3 | 3 |  | 3 | 3 |  | 3 | 3 |  | 3 | 3 |  | 0 | 0 |
| **45** | 0 | 0 |  | 0 | 0 |  | 0 | 0 |  | 0 | 0 |  | 0 | 0 |  | 0 | 0 |
| **46** | 1 | 1 |  | 1 | 1 |  | 1 | 1 |  | 2 | 2 |  | 2 | 2 |  | 1 | 1 |
| **47** | 0 | 0 |  | 0 | 0 |  | 0 | 0 |  | 0 | 0 |  | 0 | 0 |  | 0 | 0 |
| **48** | 0 | 0 |  | 0 | 0 |  | 0 | 0 |  | 0 | 0 |  | 0 | 0 |  | 0 | 0 |
| **49** | 1 | 2 |  | 2 | 2 |  | 1 | 1 |  | 2 | 2 |  | 2 | 2 |  | 0 | 0 |
| **50** | 3 | 3 |  | 3 | 3 |  | 3 | 3 |  | 3 | 3 |  | 2 | 3 |  | 0 | 0 |
| **51** | 0 | 0 |  | 0 | 0 |  | 0 | 0 |  | 0 | 0 |  | 0 | 0 |  | 0 | 0 |
| **52** | 0 | 0 |  | 1 | 1 |  | 0 | 0 |  | 1 | 2 |  | 1 | 1 |  | 0 | 0 |
| **53** | 2 | 3 |  | 2 | 3 |  | 0 | 1 |  | 2 | 2 |  | 2 | 2 |  | 0 | 0 |
| **54** | 3 | 3 |  | 3 | 3 |  | 0 | 1 |  | 3 | 3 |  | 3 | 3 |  | 0 | 0 |
| **55** | 2 | 2 |  | 2 | 2 |  | 0 | 0 |  | 2 | 1 |  | 2 | 2 |  | 0 | 0 |
| **56** | 0 | 0 |  | 0 | 1 |  | 0 | 0 |  | 2 | 2 |  | 1 | 1 |  | 0 | 0 |
| **57** | 0 | 0 |  | 0 | 0 |  | 0 | 0 |  | 0 | 0 |  | 0 | 0 |  | 0 | 0 |
| **58** | 2 | 3 |  | 2 | 3 |  | 1 | 0 |  | 2 | 2 |  | 2 | 2 |  | 0 | 0 |
| **59** | 3 | 3 |  | 3 | 3 |  | 2 | 2 |  | 3 | 2 |  | 1 | 1 |  | 0 | 0 |
| **60** | 1 | 1 |  | 1 | 1 |  | 0 | 0 |  | 2 | 2 |  | 2 | 2 |  | 0 | 0 |
| **61** | 0 | 0 |  | 1 | 1 |  | 0 | 0 |  | 2 | 2 |  | 1 | 1 |  | 0 | 0 |
| **62** | 3 | 3 |  | 3 | 3 |  | 3 | 3 |  | 3 | 2 |  | 1 | 1 |  | 0 | 0 |
| **63** | 0 | 0 |  | 0 | 0 |  | 0 | 0 |  | 0 | 0 |  | 0 | 0 |  | 0 | 0 |
| **64** | 3 | 3 |  | 3 | 3 |  | 0 | 0 |  | 3 | 3 |  | 2 | 2 |  | 0 | 0 |
| **65** | 1 | 1 |  | 1 | 1 |  | 0 | 1 |  | 1 | 2 |  | 1 | 2 |  | 0 | 0 |
| **66** | 3 | 3 |  | 3 | 3 |  | 0 | 0 |  | 3 | 3 |  | 2 | 2 |  | 0 | 0 |
| **67** | 0 | 0 |  | 0 | 0 |  | 0 | 0 |  | 0 | 0 |  | 0 | 0 |  | 0 | 0 |
| **68** | 1 | 1 |  | 1 | 1 |  | 0 | 0 |  | 2 | 1 |  | 1 | 2 |  | 0 | 0 |
| **69** | 0 | 0 |  | 0 | 0 |  | 0 | 0 |  | 2 | 2 |  | 1 | 1 |  | 0 | 0 |
| **70** | 1 | 2 |  | 1 | 2 |  | 0 | 0 |  | 2 | 1 |  | 1 | 1 |  | 0 | 0 |
